# Clinical benefits of MRI-guided freehand biopsy of small focal liver lesions in comparison to CT guidance

**DOI:** 10.1007/s00330-024-10623-9

**Published:** 2024-02-06

**Authors:** Vanessa F. Schmidt, Osman Öcal, Viktoria Walther, Matthias P. Fabritius, Olaf Dietrich, Philipp M. Kazmierczak, Lena Weiss, Sinan Deniz, Muzzafer R. Ümütlü, Daniel Puhr-Westerheide, Moritz Wildgruber, Jens Ricke, Max Seidensticker

**Affiliations:** 1 Department of Radiology, University Hospital, LMU Munich, Munich, Germany; 2 Department of Medicine III, University Hospital, LMU Munich, Munich, Germany

**Keywords:** Magnetic resonance–guided interventional procedures, Interventional magnetic resonance imaging, Biopsy (Needle), Puncture biopsy

## Abstract

**Objectives:**

To compare clinical success, procedure time, and complication rates between MRI-guided and CT-guided real-time biopsies of small focal liver lesions (FLL) < 20 mm.

**Methods:**

A comparison of a prospectively collected MRI-guided cohort (*n* = 30) to a retrospectively collected CT-guided cohort (*n* = 147) was performed, in which patients underwent real-time biopsies of small FLL < 20 mm in a freehand technique. In both groups, clinical and periprocedural data, including clinical success, procedure time, and complication rates (classified according to CIRSE guidelines), were analyzed. Wilcoxon rank sum test, Pearson’s chi-squared test, and Fisher’s exact test were used for statistical analysis. Additionally, propensity score matching (PSM) was performed using the following criteria for direct matching: age, gender, presence of liver cirrhosis, liver lobe, lesion diameter, and skin-to-target distance.

**Results:**

The median FLL diameter in the MRI-guided cohort was significantly smaller compared to CT guidance (*p* < 0.001; 11.0 mm vs. 16.3 mm), while the skin-to-target distance was significantly longer (*p* < 0.001; 90.0 mm vs. 74.0 mm). MRI-guided procedures revealed significantly higher clinical success compared to CT guidance (*p* = 0.021; 97% vs. 79%) as well as lower complication rates (*p* = 0.047; 0% vs. 13%). Total procedure time was significantly longer in the MRI-guided cohort (*p* < 0.001; 38 min vs. 28 min). After PSM (*n* = 24/*n* = 38), MRI-guided procedures still revealed significantly higher clinical success compared to CT guidance (*p* = 0.039; 96% vs. 74%).

**Conclusion:**

Despite the longer procedure time, freehand biopsy of small FLL < 20 mm under MR guidance can be considered superior to CT guidance because of its high clinical success and low complication rates.

**Clinical relevance statement:**

Biopsy of small liver lesions is challenging due to the size and conspicuity of the lesions on native images. MRI offers higher soft tissue contrast, which translates into a higher success of obtaining enough tissue material with MRI compared to CT-guided biopsies.

**Key Points:**

• *Image-guided biopsy of small focal liver lesions (FLL) is challenging due to inadequate visualization, leading to sampling errors and false-negative biopsies*.

• *MRI-guided real-time biopsy of FLL* < *20 mm revealed significantly higher clinical success (p* = *0.021; 97% vs. 79%) and lower complication rates (p* = *0.047; 0% vs. 13%) compared to CT guidance.*

• *Although the procedure time is longer, MRI-guided biopsy can be considered superior for small FLL* < *20 mm.*

**Graphical Abstract:**

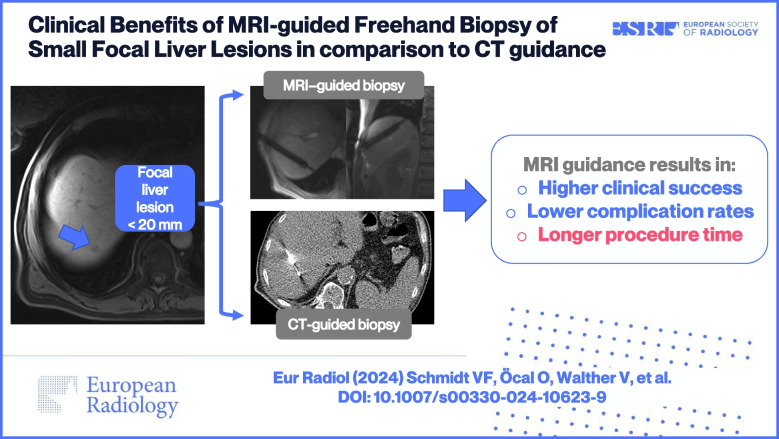

**Supplementary Information:**

The online version contains supplementary material available at 10.1007/s00330-024-10623-9.

## Introduction

Image-guided biopsy of focal liver lesions (FLL) is fundamental for an accurate diagnosis and treatment strategy, particularly in case of suspicious malignancy or known primary tumor with suspected recurrence or metastasis [1, 2]. Although the developments in diagnostic imaging in the use of multislice computed tomography (CT) or magnetic resonance imaging (MRI) with liver-specific contrast medium provide a high sensitivity regarding the differentiation of FLL [3], additional histopathological evaluation is often obligatory. Emphasized by increasing evidence of prognostic and therapeutic information obtained from histopathological and comprehensive genomic profiling, this allows identifying patients who may benefit most from targeted therapies [4, 5].

Ultrasound (US) and CT are the most commonly used modalities for liver biopsy [6, 7], although both have certain clinical limitations: US guidance may be difficult due to a restricted sonic window [8]. In the use of CT guidance, lesion conspicuity in the non-enhanced phase may be limited, and contrast agents improve lesion visualization only for a short interval of time. MRI guidance has been successfully established as an alternative for image-guided biopsy in different anatomical organs [9–12]; however, due to restricted patient access in conventional MRI systems, high costs of specialized interventional MRI systems, and longer procedure times, it is not widely used yet [13]. Nevertheless, MRI guidance offers some relevant advantages such as the non-ionizing nature of this approach, the excellent soft tissue contrast and high resolution, and the multiplanar capabilities allowing various access routes [14]. A few studies have described clinical success rates of approximately 90% of MRI-guided biopsies [15–17]. Additionally, it has been reported that in the use of CT guidance, up to 45% of small hepatic lesions were inadequately visualized with consecutive sampling errors and false-negative biopsies [18]. In contrast, using MRI guidance and liver-specific contrast agent, accurate visualization of small hepatic lesions can be exploited. To date, there is a lack of studies providing a direct comparison of both guidance modalities (MRI and CT).

In this trial, we aimed to analyze the efficacy of real-time liver biopsy in small FLL < 20 mm with respect to clinical success and complication rates by comparing a prospectively collected MRI-guided with a retrospectively collected CT-guided cohort, in general, and by propensity score matching (PSM).

## Methods

This cohort trial is approved by the Institutional Review Board of the University Hospital, LMU Munich (Protocol No.: 19–976). Both research informed consent and procedural informed consent were obtained from the prospectively collected MRI-guided cohort, whereas there was waived consent for research regarding the CT-guided cohort.

### MRI-guided patient cohort

Between November 2020 and March 2023, a total of 30 patients with at least one FLL showing a diameter < 20 mm were referred to our department for MRI-guided percutaneous liver biopsy to obtain diagnostic confirmation of suspicious liver lesions. All patients presented with known malignancy with suspected recurrence or metastasis. Indications for MRI guidance were small lesion size, suspected poor lesion visibility in other modalities, unfavorable lesion location such as in the hepatic dome, or negative results of previous biopsy procedures using US or CT guidance (see Supplemental Table 1).

All procedures were performed using a closed whole-body 1.5 T (T) scanner (Magnetom Aera; Siemens Healthineers) with a short bore design (system length cover-to-cover 145 cm, bore diameter 70 cm) and a maximum gradient strength of 45 mT/m and a slew rate of 200 T/m/s. A receive-type surface loop coil (Siemens Healthineers) with a diameter of 110 mm, allowing an open approach to the liver, was placed over the area of interest for periprocedural imaging. Next to the MRI scanner, the MRI-compatible liquid–crystal display monitor (InroomViewingDevice LCD 3.1, NordicNeuroLab) was installed for real-time monitoring. A freehand, coaxial technique was performed in all biopsies by one experienced interventional radiologist. A coaxial system with a 90 or 140 mm, 16-gauge MRI-compatible coaxial needle (ITP Innovative Tomography Products GmbH) and a 150 or 200 mm, 18-gauge semi-automated biopsy needle (ITP Innovative Tomography Products GmbH) was used to obtain lesion samples.

**Fig. 1:**
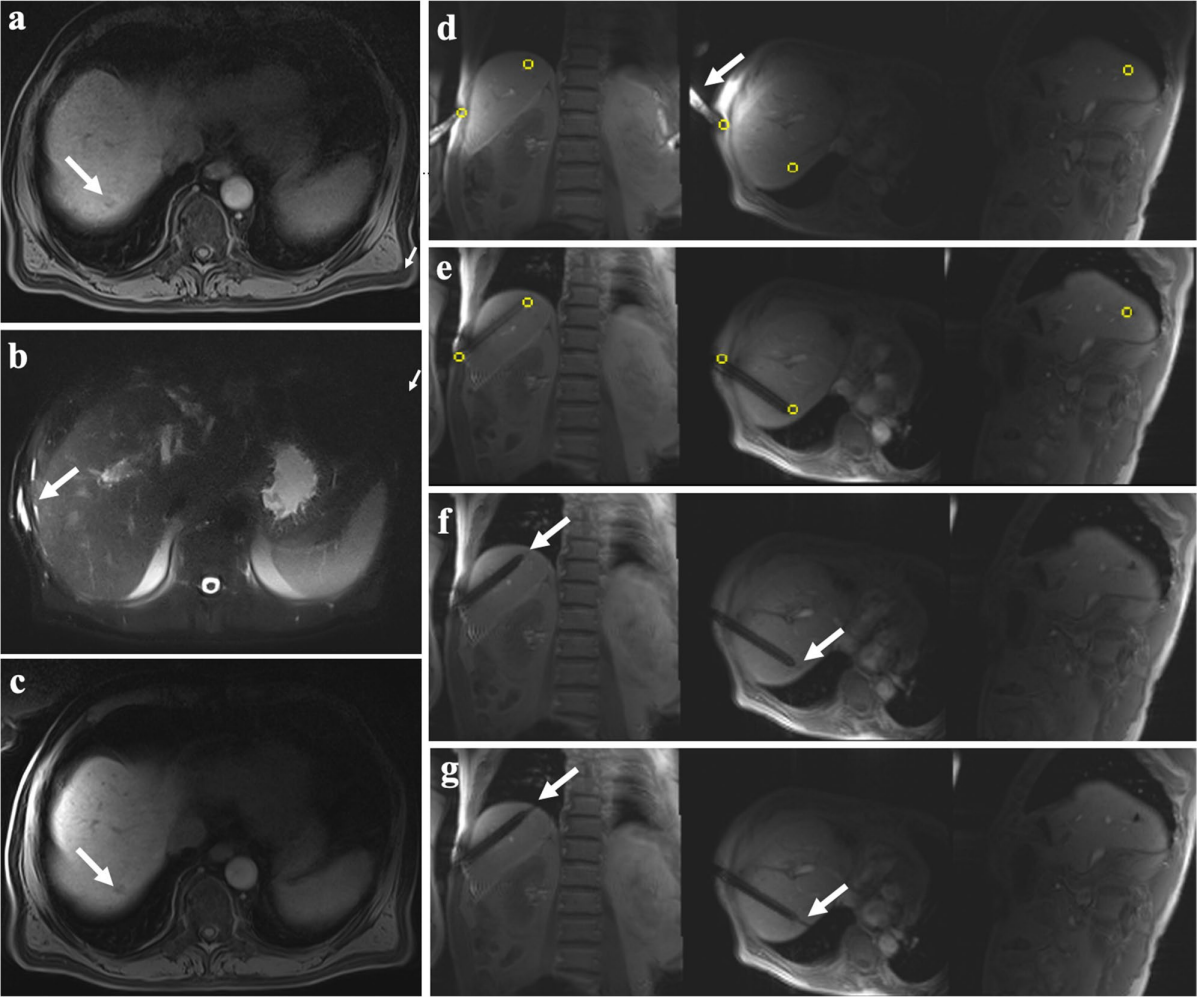
A 67-year-old male patient with known HCC and new suspected FLL located in liver segment 8 undergoing MRI-guided liver biopsy with an intercostal approach. **a** Preprocedural axial T1w GRE sequence after liver-specific contrast agent application revealed a small target lesion at the hepatic dome with a diameter of 7 mm. **d** Defining and digital marking of entry point and target lesion (yellow tags) for procedure planning in use of three-dimensional real-time T1w GRE sequences and finger-pointing (arrow). **e** Three-dimensional real-time T1w GRE sequences confirmed guidance and angulation of 16-gauge coaxial needle while interactive graphical modification of the slice geometry. **f** After fading out the yellow tags, the three-dimensional real-time T1w GRE sequences revealed the exact position of the needle tip at the lesion border (arrows). **g** Firing of the biopsy system under real-time monitoring of three-dimensional T1w GRE sequences.** b** Postprocedural axial T2w fat-saturated TSE sequences excluded relevant bleeding or subcapsular hematoma. **c** Postprocedural axial T1w GRE sequences showed two thin hypodense lines within the small FLL, compatible with matching puncture tracts

**Fig. 2:**
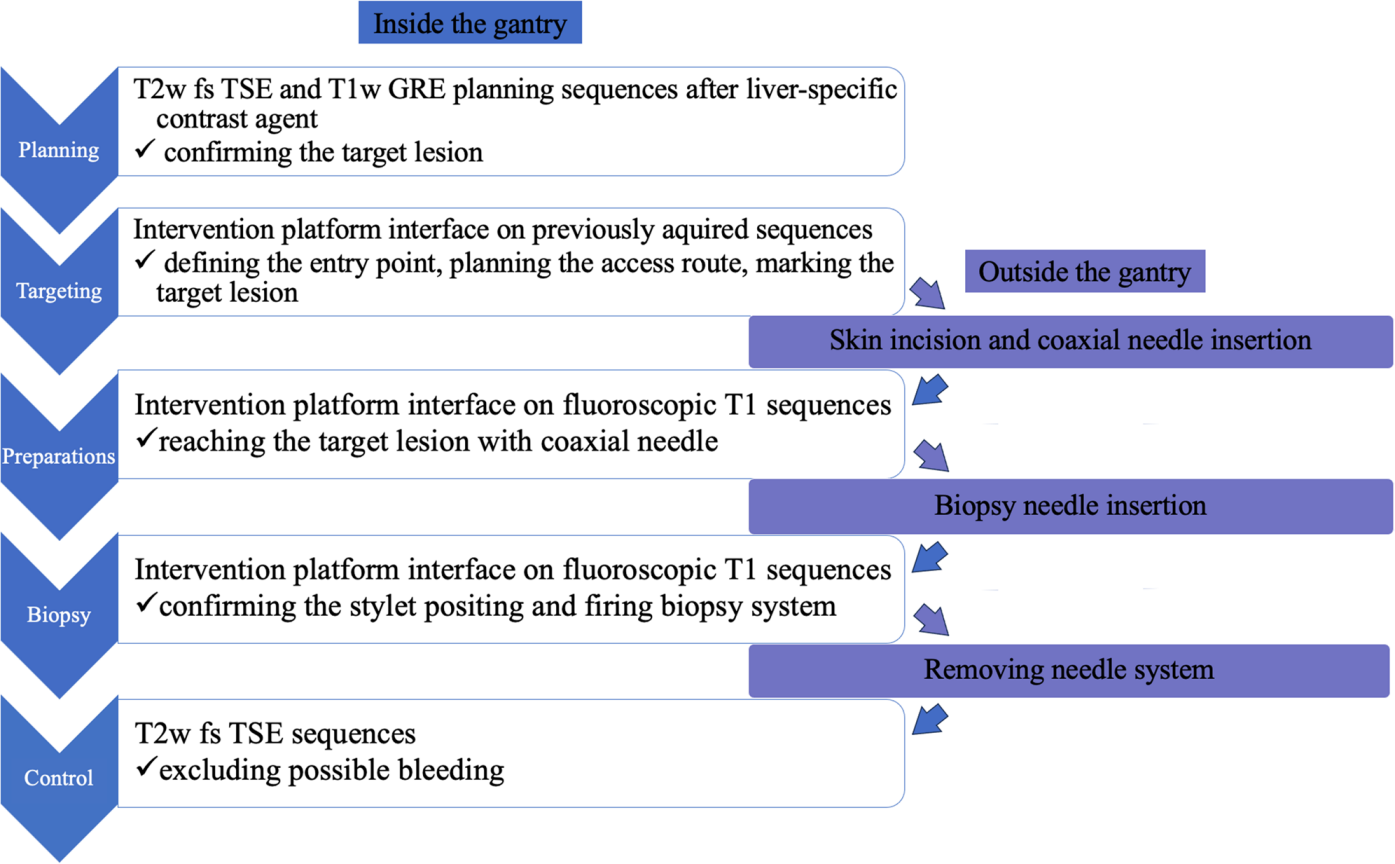
Schematic flowchart of the sequential steps during MRI-guided liver biopsy

MRI-guided biopsies were performed in local anesthesia. In all patients, after liver-specific contrast agent (1.0 mL/10 kg of Gd-EOB-DTPA, Primovist, Bayer Vital) has been injected [19], axial and coronal T1-weighted (T1w) Dixon gradient echo (GRE) sequences were used (TR 6.8 ms, TE 2.4 and 4.8 ms, flip angle 10°, slice thickness 3 mm, field of view 380 × 380 mm^2^, matrix 320 × 195, bandwidth 470 Hz/pixel, breath-hold) to confirm the target lesion and plan a safe access route using the WIP package ASP 1428B “LaserToTarget” (Siemens Healthineers). The WIP package provided an interface to localize the needle entry point on the patient (defined on previously acquired MR images) by moving this point under the built-in laser marker (that is usually used for isocentering of the patient). After defining the skin entry point with finger-pointing as well as skin marking and disinfection, sterile draping and local anesthesia were administered. Following skin incision and coaxial needle insertion, the patient was moved to the isocenter of the magnet. An MRI fluoroscopic T1w GRE sequence (WIP package ASP 1075H “Needle AutoAlign,” Siemens Healthineers; TR 8.4 ms, TE 4.5 ms, flip angle 30°, slice thickness 10 mm, field of view 320 × 320 mm^2^, matrix 128 × 128, bandwidth 250 Hz/pixel, no phase oversampling, no breath-hold or respiration compensation trigger, acquisition time: 1 frame/second) was used for image-guidance. The WIP package allowed precise digital selection of entry points and target lesions, double-oblique needle orientation, visual real-time update in three orthogonal planes, and periprocedural interactive graphical modification of the slice geometry; see Fig. 1. After reaching the target lesion and checking the needle position in three orthogonal planes, the biopsy needle was coaxially inserted outside the scanner. Moved again to the center, the stylet position was confirmed using further real-time monitoring, and the biopsy system was fired. Two to six core samples were taken from the FLL while the needle tip position was not routinely altered to sample different parts of the lesion; however, the operator was free to readjust the needle according to fluoroscopy images. The samples were fixed in 10% formalin for pathological examination. Before and after biopsy, axial T2w fat-saturated turbo spin echo (TSE) sequences (TR 800 ms, TE 85 ms, flip angle 150°, slice thickness 6 mm, field of view 380 × 380 mm^2^, matrix 320 × 320, bandwidth 710 Hz/pixel, breath-hold) were acquired for detection of possible bleeding. The intervention time contained needle insertion and obtainment of biopsy samples, which lasted from the first real-time T1w GRE sequences, including finger-pointing, until the last image showing the biopsy system. Total procedure time included set-up and move-out of equipment and patient. The sequential steps of the described procedure are summarized in a schematic flowchart; see Fig. 2.

### CT-guided patient cohort

The institutional Radiological Information System (RIS) was searched for procedures coded as “CT-guided biopsy of the liver.” From a resulting consecutive patient list of a five-year interval, 147 patients who had undergone CT-guided biopsy of small focal liver lesions (FLL) < 20 mm were retrospectively selected. All CT-guided biopsies were performed by various experienced interventional radiologists on a 128-row scanner (Somatom AS + or Edge, Siemens Healthineers) with CT fluoroscopy (CARE Vision CT, Siemens Healthineers). Depending on the phase in which the lesion is the best visible, planning CT with or without contrast media was obtained. After skin disinfection, application of local anesthesia, and sterile draping, a 16- or 18-gauge coaxial needle was advanced to the lesion under CT fluoroscopy guidance. Following this, similar to MRI-guided biopsies, two to six core samples were obtained and fixed in 10% formalin. After the removal of the needle, native CT images were obtained to evaluate possible complications. 

### Clinical success

Biopsy procedures were defined as clinically successful when a definitive histologic diagnosis could be made. In cases where the samples did not show malignant histology, e.g., inflammatory changes or other benign liver lesions, definitive histology had to be confirmed by surgery or long-term imaging follow-up (at least 6 months after biopsy) to consider the biopsy clinically successful.

### Complications

All patients underwent a biopsy as an in-house procedure with one night observation in hospital. Coagulation status (thrombocytes > 50,000/µL, INR < 1.5) and absence of contraindications to enhanced MRI or CT had been screened and standardized before the procedures. During postprocedural hospitalization, all patients were observed for potential complications, including close electronic vital sign monitoring (blood pressure, heart rate, oxygen saturation) for 2 h. In parallel, we routinely performed abdominal US imaging to exclude possible hematoperitoneum. For detection of possible subclinical bleeding, hematocrit levels were checked 4 h postprocedural. All signs of complications were classified as major and minor according to the CIRSE classification [20].

### Statistical analysis

Descriptive statistics were used to analyze the distribution of patients among the different categories. Kolmogorov–Smirnov (K-S) test was used for the assessment of normality. Data are presented as means (± standard deviation) in the case of normal distribution or as medians (interquartile range) for skewed distribution. To compare the MRI- and CT-guided cohort, Wilcoxon rank sum test, Pearson’s chi-squared test, and Fisher’s exact test were used. Statistical analysis was performed by using SPSS 26.0 statistical software (IBM SPSS Statistics). *p* < 0.05 was considered significant. Additionally, propensity score matching (PSM) was performed using R version 4.0.5, ‘‘MatchIt’’ package (version 4.9–7) [21]. Groups were matched in a 1:2 ratio, with the nearest calculated propensity logit, with a caliper width of #0.20 of the SD of the propensity score logit. The criteria for direct matching, chosen due to potential impact on the technical success, were age, gender, presence of liver cirrhosis, liver lobe, lesion diameter, and skin-to-target distance.

## Results

### Patient characteristics of MRI- and CT-guided cohorts (*n* = 30/ *n* = 147)

In the MRI-guided cohort, 14/30 (46.7%) patients were male, and the median age was 68 years (IQR 51–74). In the CT-guided cohort, 79/147 (53.7%) patients were male, and the median age was 64 years (IQR 54–70). Lesion localization was mostly in the right liver lobe in both cohorts, in 22/30 (73%) cases as well as in 93/147 (63%) cases, respectively. Furthermore, liver cirrhosis was found in 6/30 (20%) and 25/147 (17%), respectively. The most common liver pathologies in the MRI-guided cohort were metastases of colorectal cancer (mCRC), breast cancer, and malignant melanoma, each with 5/30 cases (16.7%). In the CT-guided cohort, frequent pathologies were metastases of CRC in 49/147 patients (33.3%), hepatocellular carcinoma (HCC) in 18/147 patients (12.2%), and neuroendocrine tumor (NET) metastases in 15/147 patients (10.2%). Further clinical characteristics, including all types of occurred liver pathologies and their exact distribution in both cohorts, are summarized in Table 1.

**Table 1:** Clinical characteristics of MRI- and CT-guided cohort

	MRI-guided (*n* = 30)		CT-guided (*n* = 147)		
	Number	Median (IQR)	Number	Median (IQR)	*p* value^1^
Age		68 (51–74)		64 (54–70)	> 0.9
Male	14 (46.7%)		79 (53.7%)		0.5
Liver pathology	30 (100%)		147 (100%)		> 0.9
mCRC	5 (16.7%)		49 (33.3%)		
Breast cancer metastases	5 (16.7%)		14 (9.5%)		
Malignant melanoma metastases	5 (16.7%)		13 (8.8%)		
Pancreatic cancer metastases	2 (6.7%)		11 (7.5%)		
NET metastases	3 (10.0%)		15 (10.2%)		
Adrenocortical cancer metastases	1 (3.3%)		0 (0.0%)		
Renal cell cancer metastases	1 (3.3%)		2 (1.4%)		
Liposarcoma	0 (0.0%)		2 (1.4%)		
Lymphoma	0 (0.0%)		2 (1.4%)		
Prostate cancer metastases	0 (0.0%)		2 (1.4%)		
HCC	2 (6.7%)		18 (12.2%)		
Regernative liver nodule	3 (10.0%)		7 (4.8%)		
Other non-malignant lesion^2^	3 (10.0%)		12 (8.2%)		
Liver lobe					0.3
Right	22 (73.3%)		93 (63.3%)		
Left	8 (26.7%)		54 (36.7%)		
Liver cirrhosis	6 (20.0%)		25 (17.0%)		0.8

^**1**^ Wilcoxon rank sum test; Pearson’s chi-squared test; Fisher’s exact test

^**2**^ Histological finding of inflammation or benign liver lesion confirmed by imaging in the long term (up to at least 6 months post-biopsy)

*mCRC*, metastases of colorectal cancer; *HCC*, hepatocellular carcinoma; *IQR*, interquartile range; *NET*, neuroendocrine tumor

### Comparison between MRI- and CT-guided cohort (*n* = 30 /*n* = 147)

While all FLL were < 20 mm, the median diameter in the MRI-guided cohort was significantly smaller in comparison to the CT-guided cohort (*p* < 0.001; 11.0 mm vs. 16.3 mm). Additionally, the skin-to-target distance was significantly longer in the MRI-guided cohort (*p* < 0.001; 90.0 mm vs. 74.0 mm). 

MRI-guided procedures revealed a significantly higher clinical success rate compared to CT guidance (*p* = 0.021; 29/30, 97% vs. 116/147, 79%) as well as a significantly lower complication rate (*p* = 0.047, 0/30, 0% vs. 19/147, 13%), as there were zero complications during or after MR guidance. The minor complications in the CT-guided cohort were subcapsular liver hematoma (16/147, 11%), air trapping in the anterior mediastinum (1/147, 1%), and a small pneumothorax (1/147, 1%) that regressed spontaneously. The major complication in 1/147 (1%) CT-guided case was an active hepatic artery bleeding in segment 6, resulting in a rupture of the caudal liver capsule, resolved by angiographical embolization. Total procedure time was significantly longer in the MRI-guided cohort than in the CT-guided cohort (*p* < 0.001; 38 min vs. 28 min). Details of these analyses are shown in Table 2.

**Table 2:** Comparison between MRI- and CT-guided biopsy cohort

	MRI-guided (*n* = 30)		CT-guided (*n* = 147)		
	Number	Median (IQR)	Number	Median (IQR)	*p* value^1^
Technical success^2^	30 (100%)		147 (100%)		> 0.9
Clinical success^3^	29 (96.7%)		116 (78.9%)		0.021
Lesion diameter (mm)		11.0 (9.0–12.8)		16.3 (12.5–18.2)	< 0.001
Skin-to-target distance (mm)		90.0 (75.3–102.8)		74.0 (62.2–88.9)	< 0.001
Complications	0 (0%)		19 (12.9%)		0.047
Major complications	0 (0%)		1 (0.7%)		
Minor complications	0 (0%)		18 (12.2%)		
Total procedure time (min)		38 (33–46)		28 (23–39)	< 0.001

^1^ Wilcoxon rank sum test; Pearson’s chi-squared test; Fisher’s exact test

^2^At least one tissue sample obtained

^3^Definitive histologic diagnosis could be made

*IQR*, interquartile range

### Additional validation (PSM of MRI- and CT-guided cohort, *n* = 24/ *n* = 38)

After PSM, according to the selected matching criteria, MRI-guided procedures still revealed significantly higher clinical success rate compared to CT guidance (*p* = 0.039; 23/24, 96% vs. 28/38, 74%). Regarding the complication rate, there was no significant difference between both cohorts after PSM (*p* = 0.2; 0/24, 0% vs. 4/38, 11%).

Total procedure time was significantly longer in the MRI-guided cohort than in the CT-guided cohort (*p* = 0.008; 39 min vs. 27 min). Details of these analyses are shown in Table 3.

**Table 3:** Propensity score matching of MRI- and CT-guided cohort

	MRI-guided (*n* = 24)		CT-guided (*n* = 38)		
	Number	Median (range)	Number	Median (range)	*p* value^1^
Technical success^2^	24 (100%)		38 (100%)		> 0.9
Clinical success^3^	23 (95.8%)		28 (73.7%)		0.039
Lesion diameter (mm)		11.0 (9.8–14.0)		12.9 (9.8–15.9)	0.5
Skin-to-target distance (mm)		86.0 (73.8–98.8)		74.5 (67.0–98.5)	0.3
Complications	0 (0%)		4 (10.5%)		0.2
Major complications	0 (0%)		0 (0%)		> 0.9
Minor complications	0 (0%)		4 (10.5%)		0.2
Total procedure time (min)		39 (19–65)		27 (18–70)	0.008

^1^ Wilcoxon rank sum test; Pearson’s chi-squared test; Fisher’s exact test

^2^At least one tissue sample obtained

^3^Definitive histologic diagnosis could be made

*IQR*, interquartile range

## Discussion

In this study, we compared a prospectively enrolled cohort of 30 MRI-guided biopsies of FLL < 20 mm with a retrospectively collected CT-guided cohort of 147 cases. This direct comparison regarding freehand liver biopsy of small target lesions showed the superiority of MRI guidance in terms of clinical success and complication rate.

A previous work by Stattaus et al already reported that the conspicuity of small liver lesions may diminish significantly during CT-guided biopsy due to needle artifacts, leading to an increased rate of insufficiently visualized lesions and subsequently false-negative histological results [18]. In addition, contrast enhancement during CT guidance did not reveal better results as the visibility was only temporarily improved, with complete obscuration in the late phase [18]. The clinical success of CT-guided biopsy of FLL < 30 mm was reported to be 86% [18], which is comparable to the clinical success of 79% in the CT-cohort in our study with FLL < 20 mm. In contrast, there are also reports of higher rates of clinical success after CT-guided liver biopsy in small liver lesions, for example, in a large series by Ma et al [22], who compared CT- and US guidance in FLL < 30 mm, finding clinical success rates of approximately 95% in both groups, which is similar to the results in our MRI-guided cohort. It should be noted that in the CT-guided cohort of Ma et al, both core biopsy technique and fine needle aspiration were used in combination. Furthermore, selection bias in favor of each imaging method might have been present due to the study design, as in 4% of the cases initially considered for US-guided procedures. CT was finally used because of difficulty in lesion visualization, and in contrast to our cohort, the lesion diameters up to 30 mm were still larger [22].

In the present study, the MRI-guided cohort was superior to the CT-guided cohort in terms of clinical success, although the median lesion diameter was smaller and the skin-to-lesion distance during MRI guidance was longer, which both increases the difficulty of precise lesion targeting, thus emphasizing the value of MRI guidance. In general, such selective comparison between MRI and CT guidance during biopsy of FLL, which all presented with a diameter < 20 mm, has not been performed yet. Both cohorts also presented with a similar lesion distribution in the right and left hepatic lobes. To address the slight differences between the two cohorts in our study, we additionally performed PSM while matching all clinically relevant variables potentially influencing the technical success (age, gender, presence of liver cirrhosis, liver lobe, lesion diameter, and skin-to-target distance): here, MRI-guided biopsies of FLL < 20 mm still had significantly higher clinical success rates than the CT-guided cohort, confirming the previously obtained result. On the one hand, these results may be due to the significantly better soft tissue contrast and characterization with subsequently improved visualization of FLL as well as clear identification of adjacent vascular structures and normal tissues (for example, bile ducts), which would be impossible on CT without contrast agents [17, 23]. On the other hand, needle artifacts can also be observed during MRI-guided interventions, but these are less limiting or prominent than metal artifacts during CT guidance and furthermore, can be optimized by modifying sequence parameters and the intervention angle related to B0 [24, 25].

Regarding complications, we found significant differences between the cohorts without PSM, as there were recorded no complications for MR guidance and a complication rate of 13% for CT guidance, which may also be related to the more accurate visualization of risk structures. Regarding this difference, it should be emphasized that both cohorts received identical and systematic postprocedural monitoring. This observation was similarly described in image-guided liver interventions by Li et al, reporting significantly fewer complications when comparing MRI-guided to CT-guided microwave ablation of FLL ablation (6% vs. 46%) [26]. In terms of complication rates, differences between both cohorts were also seen after PSM, with 11% versus 0%; nevertheless, this was not statistically significant, probably due to the smaller group sizes. Generally, regarding CT guidance, results with lower complication rates can also be found in the literature [27, 28]; exemplarily Stattaus et al report a complication rate of 4.3% in 163 biopsies of FLL using 16- and 18-gauge needles. However, these studies have not been restricted to small lesion diameters.

The zero-complication rate for MR guidance has been achieved despite the significantly longer procedure time, suggesting that the visualization may be more crucial than the duration of the procedure itself in order to avoid complications. In general, the longer overall procedure time is often reported as one of the known disadvantages of MR-guided interventions. While we can confirm this in our analyses, one point to consider is the longer skin-to-target distance in the MR-guided cohort, which may be at least in part responsible for longer total procedure times [29]. However, there was still a significant difference after PSM and matching the cohort to this criterion, so this was not a determining factor.

There are several limitations regarding our results that should be noted. This is a single-center cohort study comparing a prospectively enrolled group to a retrospectively collected group. The prospective group involved a small number of cases, which may have led to biased results. As the retrospective series was acquired independently of the MRI-guided cohort in terms of time, there was no selection bias regarding the chosen imaging method. Although there were differences in baseline characteristics that favored CT guidance in terms of success with larger lesions closer to the skin, MRI-guided biopsies resulted in significantly better success, which was also confirmed after eliminating differences by PSM. Nevertheless, this needs to be further evaluated in larger sample sizes and prospectively set up studies, including randomized cohort and multicenter studies, in order to gain more evidence.

In conclusion, compared with previously published studies that have reported mostly similar results of MRI and CT in individual studies regarding one of both modalities, we suggest considering freehand biopsy of small FLL < 20 mm in MR guidance even superior to CT guidance regarding the significant higher clinical success as well as lower complication rates. Although the procedure time is longer, MRI-guided biopsy may be considered primary for small lesions, as the clinically and economically insignificant median time extension of 10 min should definitely be justified by the higher clinical success rate of 15% and, consecutively, by the diagnostic benefit.

### Supplementary Information

Below is the link to the electronic supplementary material. 

Supplementary file1 (PDF 32 KB)

## Abbreviations

CIRSE: Cardiovascular and Interventional Radiological Society of Europe
CT: Computed tomography
FLL: Focal liver lesion
GRE: Gradient echo
HCC: Hepatocellular carcinoma
IQR: Interquartile range
mCRC: Metastases of colorectal cancer
MRI: Magnetic resonance imaging
NET: Neuroendocrine tumor
PSM: Propensity score matching
RIS: Radiological information system
T: Tesla
T1w: T1-weighted
T2w: T2-weighted
TE: Echo time
TR: Repetition time
TSE: Turbo spin echo
US: Ultrasound

## Declarations

### Guarantor

The scientific guarantor of this publication is Vanessa F. Schmidt.

### Statistics and biometry

One of the authors has significant statistical expertise.

### Study subjects or cohorts overlap

No study subjects or cohorts have been previously reported.

### Methodology

• prospective/retrospective

• cross-sectional study

• performed at one institution
