# Supplementary material for: Clinical benefits of MRI-guided freehand biopsy of small focal liver lesions in comparison to CT guidance
Source: Eur Radiol. 2024 Feb 6;34(9):5507–16. doi: 10.1007/s00330-024-10623-9 (PMC11364707; doi:10.1007/s00330-024-10623-9)
Supplement: Supplementary file 1 — Supplementary file1 (PDF 32 KB) [file 330_2024_10623_MOESM1_ESM.pdf]

Clinical Benefits of MRI-guided Freehand Biopsy of Small Focal Liver Lesions in comparison to CT guidance

Electronic Supplementary Material (ESM)

Eur Radiol (2024) Schmidt VF, Öcal O, Walther V et al.

Supplemental Table 1. Indications for MRI guidance of liver biopsy

	MRI-guided cohort (n=30) Number
Small lesion size <20 mm	30 (100%)
Suspected poor visibility in other modalities	8 (%)
Unfavorable lesion location (e.g. hepatic dome)	5 (16.7%)
Negative results of previous biopsy procedures using CT or US guidance	12 (40%)

[1] e.g.="exempli gratia" (lat.), for example; CT=computed tomography; US=ultrasound
